# Pharmacological Inactivation of CatSper Blocks Sperm Fertilizing Ability Independently of the Capacitation Status of the Cells: Implications for Non-hormonal Contraception

**DOI:** 10.3389/fcell.2021.686461

**Published:** 2021-07-06

**Authors:** Ludmila Curci, Guillermo Carvajal, Valeria Sulzyk, Soledad Natalia Gonzalez, Patricia S. Cuasnicú

**Affiliations:** Instituto de Biología y Medicina Experimental (IByME-CONICET), Ciudad Autónoma de Buenos Aires, Buenos Aires, Argentina

**Keywords:** sperm, egg, capacitation, hyperactivation, fertilization, contraception

## Abstract

Cation channel of sperm (CatSper), the main sperm-specific Ca^2+^ channel, plays a key role in mammalian fertilization, and it is essential for male fertility, becoming an attractive target for contraception. Based on this, in the present work, we investigated the effects of CatSper inactivation on *in vitro* and *in vivo* sperm fertilizing ability and the mechanisms underlying such effects. Exposure of cauda epididymal mouse sperm to different concentrations (1–20 μM) of the potent CatSper inhibitor HC-056456 (HC) during *in vitro* capacitation showed no effects on sperm viability but significantly affected Ca^2+^ entry into the cells, progressive motility, protein tyrosine phosphorylation, induced acrosome reaction, and hyperactivation, as well as the sperm’s ability to *in vitro* fertilize cumulus oocyte complexes and zona-free eggs. Whereas the presence of HC during gamete coincubation did not affect *in vitro* fertilization, exposure of either non-capacitating or already capacitated sperm to HC prior to gamete coincubation severely reduced fertilization, indicating that sperm function is affected by HC when the cells are incubated with the drug before sperm–egg interaction. Of note, insemination of HC-treated sperm into the uterus significantly or completely reduced the percentage of oviductal fertilized eggs showing, for the first time, the effects of a CatSper inhibitor on *in vivo* fertilization. These observations, together with the finding that HC affects sperm fertilizing ability independently of the sperm capacitation status, provide further insights on how CatSper regulates sperm function and represent a solid proof of concept for developing a male/female non-hormonal contraceptive based on the pharmacological blockage of CatSper activity.

## Introduction

Mammalian fertilization is a complex process that involves a series of orchestrated steps. After leaving the testes, spermatozoa undergo several physiological changes during their transit through the male and female reproductive tracts known as maturation (Robaire and Hinton, 2014) and capacitation (Chang, 1951; Austin, 1952), respectively. Sperm maturation occurs while sperm are passing through the epididymis and confers sperm the ability to move progressively and to recognize and fertilize the egg. This is followed by a capacitation process that takes place while sperm are ascending through the female tract toward the egg and which allows sperm to undergo both the acrosome reaction, an exocytotic event in the head, essential for both penetration of the zona pellucida (ZP) that surrounds the egg and membrane fusion with the egg plasma membrane, and hyperactivation, an extremely vigorous motility pattern with high-amplitude asymmetrical flagellar beating essential for detaching sperm from the oviductal epithelium (Demott and Suarez, 1992) and for allowing sperm to reach the ampulla (Suarez et al., 1991) and penetrate the egg coats (Stauss et al., 1995).

Although the mechanisms underlying mammalian fertilization remain to be fully elucidated, one molecule that plays a key role in this process is cation channel of sperm (CatSper), a sperm-specific, weakly voltage-dependent, Ca^2+^-selective, and pH-sensitive ion channel (Sun et al., 2017). Although CatSper is evolutionarily conserved from invertebrates (Cai et al., 2014) to mammals, so far, the only species with electrophysiologically confirmed CatSper currents are human (Lishko and Kirichok, 2010), macaque (Sumigama et al., 2015), and mouse (Kirichok et al., 2006) with regulatory mechanisms that differ significantly among species, i.e., murine CatSper is insensitive to human CatSper activators such as progesterone and prostaglandins (Lishko et al., 2011).

CatSper, the main mammalian sperm Ca^2+^ channel, is exclusively expressed in the testes, specifically localized to the principal piece of the tail (Hwang et al., 2019), and essential for hyperactivation (Carlson et al., 2003; Quill et al., 2003). Its sophisticated architecture consists in at least 10 different subunits that give rise to a heteromeric complex including four α pore-forming subunits CatSper 1 to 4 (Ren et al., 2001; Quill et al., 2003; Qi et al., 2007) and six auxiliary subunits, CatSper β, γ, δ, ε, ζ, and EFCAB9 (Liu et al., 2007; Wang et al., 2009; Chung et al., 2011; Chung et al., 2017 and Hwang et al., 2019). The disruption of any of the CatSper subunits results in channel dysfunction leading to either male infertility/subfertility without other phenotypic abnormalities (Chung et al., 2011; Ren et al., 2001; Jin et al., 2007; Qi et al., 2007; Chung et al., 2017; Hwang et al., 2019) whereas, female mice remain healthy and fertile. Accordingly, in humans, genomic mutations at different CatSper subunits (Avidan et al., 2003; Zhang et al., 2007; Avenarius et al., 2009; Brown et al., 2018; Luo et al., 2019) have been shown to cause a number of sperm abnormalities associated, in the majority of the cases, with asthenoteratozoospermia in infertile men (Avidan et al., 2003; Zhang et al., 2007; Avenarius et al., 2009). A comparison of the flagellar beating of wild type and CatSper-null spermatozoa revealed that mutant spermatozoa were unable to display hyperactivation even in capacitating media (Carlson et al., 2003; Quill et al., 2003; Jin et al., 2007). In agreement with the lack of hyperactivation development, CatSper mutant sperm showed a lower percentage of progressive motility than wild type spermatozoa in a high viscous medium (Quill et al., 2003), cannot move beyond the oviductal sperm reservoir because of insufficient mechanical force to detach from oviductal epithelial cells (Ho et al., 2009), and cannot penetrate the egg’s protective vestments during *in vitro* fertilization in mouse (Singh and Rajender, 2015) and human (Brown et al., 2018).

Given the relevance of this channel for fertilization, CatSper1–4 and/or the auxiliary subunits become potential candidates for either the development of new methods of diagnosis and/or treatment of infertility (Tamburrino et al., 2014; Wang et al., 2021) or to be targeted for contraception. In this regard, CatSper fulfills several of the requisites for a contraceptive candidate target as it localizes on the surface of mammalian sperm being accessible to drug intervention, it is exclusively expressed in the testes and sperm, avoiding potential side effects, it plays key roles in several stages of fertilization, and it is essential for male fertility. Interestingly in this regard, spermatogenesis was shown to be normal in men where CatSper function could not be detected in ejaculated spermatozoa (Williams et al., 2015), emphasizing the potentiality of this channel as a feasible contraceptive target in men. Moreover, considering that sperm are present in both the male and female reproductive tracts, CatSper might be a good target for contraception also in women. Of note in this regard, whereas knockout (KO) models have provided most of the evidence on the relevance of CatSper for fertilization/fertility, pharmacological approaches avoid the functional compensation of the lacking molecule that may occur during development of KO mice and provide important information on timing and reversibility aspects of the molecule functional role. Based on this, in the present work, we studied the effects of the pharmacological inactivation of CatSper on sperm fertilizing ability *in vitro* and *in vivo* and analyzed the mechanisms underlying such effects to both advance our understanding on how CatSper regulates sperm function and explore its potentiality as a non-hormonal contraceptive target.

## Materials and Methods

### Animals

Adult male (2–3 months old) and young adult female (1.5–4 months old) F1 hybrids of BALB/c male and C57BL/6 female mouse strains were used. Animals were maintained with food and water *ad libitum* in a temperature-controlled room with a 12:12-h light:dark cycle. Experiments were conducted in accordance with the *Guide for Care and Use of Laboratory Animals* published by the National Institutes of Health (NIH).

### Sperm Collection and *in vitro* Sperm Capacitation

Sperm were recovered from young adult males by incising cauda epididymides in 300 μl of capacitation medium (Fraser and Drury, 1975) supplemented with 0.3% of bovine serum albumin (BSA) under paraffin oil (swim out process) where they reach a final concentration of 1–10 × 10^7^ sperm/ml. For capacitation, sperm were counted and aliquots of the suspension (fresh sperm) were added to 300 μl of capacitating medium under paraffin oil to reach a final concentration of 1–10 × 10^6^ sperm/ml. Sperm suspensions were then incubated for 90–120 min at 37°C in an atmosphere of 5% CO_2_ in air in order to accomplish capacitation. To study the effects of CatSper inhibition on sperm function, sperm were exposed either before, during, or after capacitation, to different concentrations of the channel inhibitor HC-056456 (HC) [3,4-bis(2-thienoyl)-1,2,5-oxadiazole-*N*-oxide (C*12*H*6*N*2*O*4*S*2*), Mw: 306.32], identified as a potential inhibitor of CatSper in a chemical-library screen (Carlson et al., 2009), using the vehicle dimethyl sulfoxide (DMSO) as control.

### Evaluation of Sperm Functional Parameters

#### Sperm Viability

Sperm viability was assessed using a dye-exclusion technique as previously described (Ellerman et al., 1998). Briefly, 10 μl of motile sperm suspensions were stained with prewarmed eosin 0.5% (yellowish; Sigma) in saline solution placed between prewarmed slides and coverslips, and the incorporation of the dye was evaluated under a light microscope (SWIFT 7856172) at 400x. As the plasma membrane of viable spermatozoa is a barrier to dye penetration, the percentage of sperm viability was calculated as the number of sperm that did not incorporate the dye over the total number of sperm counted.

#### Sperm Progressive Motility

Sperm suspensions (15 μl) were placed between prewarmed slides and coverslips (22 mm × 22 mm) to create a chamber with ∼30 μm depth and sperm movement was recorded by video microscopy under a light microscope (Nikon ECLIPSE E200; Basler acA-78075gc 21517342 camera; pylon5 viewer) at 400x magnification for subsequent analysis. The percentage of progressive motile sperm was calculated by analyzing a minimum of 300 cells distributed in at least 20 different microscope fields.

#### Computer-Assisted Sperm Analysis (CASA)

Sperm aliquots (15 μl) were placed between prewarmed slides and coverslips (22 mm × 22 mm) to create a chamber with ∼30 μm depth and were examined at 37°C using the ISAS^®^ (Integrated Semen Analysis System) v1.2 computer-assisted sperm analysis (CASA) system (Proiser R&D, S.L., Valencia, Spain). For each sample, a minimum of 200 cells distributed in at least 20 different microscope fields were scored (30 frames acquired at 60 Hz for each measurement). Sperm were considered motile when showing straight-line velocity (VSL, μm/s) > 0, and hyperactivated when presenting curvilinear velocity (VCL, μm/s) ≥ 266, linearity (LIN, %) < 25.9, and amplitude of lateral head displacement (ALH, μm) ≥ 5. These custom cutoffs were calculated based on the values corresponding to control animals in each colony following our previous experience (Brukman et al., 2016; Carvajal et al., 2018) and recommendations (Bray et al., 2005). All CASA assays were carried out using 1 and 5 μM HC because higher HC concentrations severely affected sperm motility, preventing it from reaching the at least 200 motile sperm required for proper analysis.

#### Protein Tyrosine Phosphorylation

Sperm suspensions were washed with PBS and resuspended in Laemmli sample buffer (Laemmli, 1970). Samples were boiled for 5 min and centrifuged at 5,000 × *g*. The supernatants were recovered and boiled again in the presence of 70 mM 2-β-mercaptoethanol (Sigma). Solubilized proteins (corresponding to 0.5–1 × 10^6^ spermatozoa/lane) were separated by SDS-PAGE, transferred onto nitrocellulose, and immunoblotted with the antiphosphotyrosine monoclonal antibody (1:10,000; clone 4G10; Upstate, Lake Placid, NY, United States), as previously described (Da Ros et al., 2004).

#### Spontaneous, Ionophore-Induced, and Progesterone-Induced Acrosome Reaction

The acrosomal status of epididymal sperm capacitated in the presence of HC or DMSO (as control) was evaluated by Coomassie Brilliant Blue staining, as previously described (Visconti et al., 1999) with slight modifications. Briefly, after fixation with 1 vol of 8% paraformaldehyde in PBS, sperm were washed with 0.1 M ammonium acetate, pH: 9, mounted on slides, and air dried. Slides were washed by successive immersions in water, methanol, and water (5 min each) and then incubated in 0.22% Coomassie Brilliant Blue G250 solution (50% methanol and 10% acetic acid) for 3 min. After staining, slides were thoroughly washed with distilled water, mounted, and immediately observed to avoid diffusion of the stain. Four hundred spermatozoa were evaluated in each treatment under a light microscope (SWIFT 7856172) at × 400. Sperm were scored as acrosome-intact when a bright blue staining was observed in the dorsal region of the head or as acrosome-reacted when patchy or no labeling was observed. For induction of the acrosome reaction, sperm were exposed to 10 μM Ca^2+^ ionophore A23187 (Sigma) or 30 μM progesterone (Sigma) during the last 15 min of capacitation. For evaluation of the spontaneous acrosome reaction, capacitated sperm were exposed only to DMSO.

#### Intracellular Ca^2+^

Intracellular Ca^2+^ levels were measured by flow cytometry, as previously described (June et al., 1997). Briefly, after 60 min of incubation in capacitation medium, sperm were loaded with 2 mM of Fluo-4 AM (Invitrogen, Carlsbad, CA, United States) diluted in 10% (*w*/*v*) Pluronic F-127 (Invitrogen, Carlsbad, CA, United States) during a 30-min incubation in the presence of the probe. Samples were washed to remove the excess of the probe, resuspended in BSA-free medium, and exposed to 2.5 μg/ml propidium iodide (PI, Sigma-Aldrich, San Louis, MO, United States). Fluorescence was detected using a BD FACSCantoTM II analyzer following the manufacturer’s indications. Data analysis was performed by FlowJo 7.6 software (FlowJo LLC, Ashland, OR, United States). Results are shown as mean fluorescence intensity (MFI) for Fluo-4 AM obtained from at least 10,000 live (PI negative) sperm.

### *In vitro* Fertilization Assays

Female mice were superovulated by intraperitoneal injection (i.p.) of equine chorionic gonadotropin (5 UI; Syntex, Buenos Aires, Argentina), followed by i.p. administration of human chorionic gonadotropin (5 UI; Syntex, Buenos Aires, Argentina) 48 h later. Oocyte-cumulus complexes (COC) were collected from oviducts 12–15 h after human chorionic gonadotropin administration and were coincubated with capacitated sperm (final concentration: 0.5–2 × 10^5^ sperm/ml) for 3 h under paraffin oil at 37°C in an atmosphere of 5% CO_2_ in air. COC were then washed, fixed, stained with 1 μg/ml Hoechst 33342, evaluated under an Optiphot microscope (Nikon) using a × 20 NA 0.50 objective, and the percentages of fertilized eggs were determined. Eggs were considered fertilized when at least one decondensing sperm nucleus or two pronuclei were observed in the egg cytoplasm.

### Evaluation of Gamete Fusion

Cumulus complexes were collected from the oviducts as described above. Cumulus cells were removed by incubating the COC for 3 min in 0.3 mg/ml hyaluronidase (type IV; Sigma), and the zona pellucida (ZP) was dissolved by treating the oocytes with acid Tyrode solution (pH 2.5) for 10–20 s (Nicolson et al., 1975). The obtained ZP-free oocytes were inseminated with capacitated sperm (final concentration: 1–5 × 10^4^ sperm/ml) and gametes were coincubated for 60 min under paraffin oil at 37°C in an atmosphere of 5% CO_2_ in air. Oocytes were then washed in fresh medium to remove loosely adherent sperm, stained with Hoechst 33342 (10 μg/μl), examined under a fluorescent Optiphot microscope (Nikon), and the percentages of fertilized eggs were determined. Eggs were considered fertilized when at least one decondensing sperm nucleus was observed in the egg cytoplasm.

### Intrauterine Insemination

Seven hours after human chorionic gonadotropin administration, females were anesthetized with ketamine (100 mg/kg, Holliday-Scott SA, Buenos Aires, Argentina)—xilacine (10 mg/kg, Richmond Vet Farma SA, Buenos Aires, Argentina), and both uterine horns were surgically exposed. Then, sperm suspensions at a final high, non-capacitating concentration (1–10 × 10^7^ sperm/ml, 50 μl) were preincubated with HC (or DMSO) for different times and injected into one of the uterine horns using the contralateral horn as control. After insemination, the reproductive organs were replaced into the body cavity, body wall and skin were stitched, and females were then placed on a warm pad in a clean cage to recover. After 15 h, oocytes were recovered from the ampulla and the percentage of fertilized eggs was analyzed by staining with Hoechst as described above.

### Statistical Analysis

Data represent the mean ± SEM from at least three independent experiments. Calculations were performed using the Prism 6.0 software (GraphPad Software, La Jolla, CA, United States). Most comparisons were made by one-way ANOVA followed by Bonferroni posttest vs. control. Motility time course studies were evaluated by two-way ANOVA and Bonferroni posttest, and intrauterine insemination assays were analyzed by Student’s *t*-test for each timepoint. Differences were considered significant at a level of *p* < 0.05.

## Results

### Effects of HC on Capacitation-Associated Sperm Functional Parameters

**FIGURE 1:**
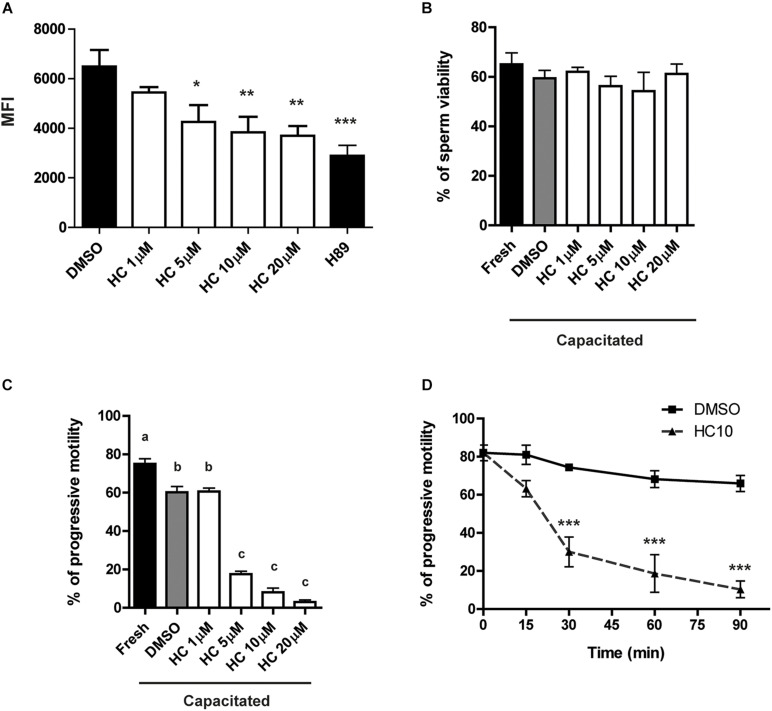
Effect of HC on sperm intracellular Ca^2+^ levels and progressive motility during capacitation. Cauda epididymal sperm were incubated for 90 min under capacitating conditions in medium containing either different concentrations of HC (1, 5, 10, and 20 μM) or DMSO (vehicle) as a control, and then subjected to different analysis. **(A)** Intracellular Ca^2+^ levels determined by flow cytometry using Fluo 4-AM and PKA inhibitor H89 as a positive control of Ca^2+^ entry inhibition. Results are shown as mean fluorescence intensity (MFI). **(B)** Percentage of sperm viability determined by eosin staining. **(C)** Percentage of sperm progressive motility determined by light microscopy (× 400). Values not sharing the same letter are significantly different. **(D)** Percentage of sperm progressive motility as a function of incubation time in a medium containing HC 10 μM. Samples were analyzed as described in panel **(C)**. Data are mean ± SEM of at least *n* = 3 independent experiments; *p < 0.05; **p < 0.005; ***p < 0.0005 vs. DMSO.

Previous results showed that exposure of mouse sperm to HC, an efficient CatSper blocker, significantly inhibits CatSper currents (Carlson et al., 2009; Ernesto et al., 2015). Based on this, and considering that CatSper is the principal sperm Ca^2+^ channel, we first analyzed whether HC was capable of inhibiting the characteristic Ca^2+^ increase that takes place during sperm capacitation (Ruknudin and Silver, 1990). For this purpose, sperm were incubated for 90 min under capacitating conditions in the presence of either different concentrations of HC (1, 5, 10, and 20 μM), DMSO (vehicle) as negative control, or H89 (a PKA inhibitor) as a positive control of Ca^2+^ entry inhibition. At the end of incubations, Ca^2+^ levels were measured by flow cytometry using the probe Fluo 4-AM. Results showed that exposure of sperm to HC produced a significant decrease in mean fluorescence intensity compared with controls which began at 5 μM and reached maximum levels at 10 μM (Figure 1A). Whereas HC did not affect sperm viability at any of the concentrations tested (Figure 1B), it produced a significant and concentration-dependent reduction in the percentage of progressive sperm motility at concentrations ≥ 5 μM as revealed by both light microscopy (Figure 1C) and CASA (Supplementary Figure 1). A subsequent time-course study using a fixed concentration of 10 μM HC during capacitation showed that progressive motility inhibition became evident at 30 min, reaching levels lower than 5% (from 80% initial motility) at the end of the incubation (Figure 1D).

**FIGURE 2:**
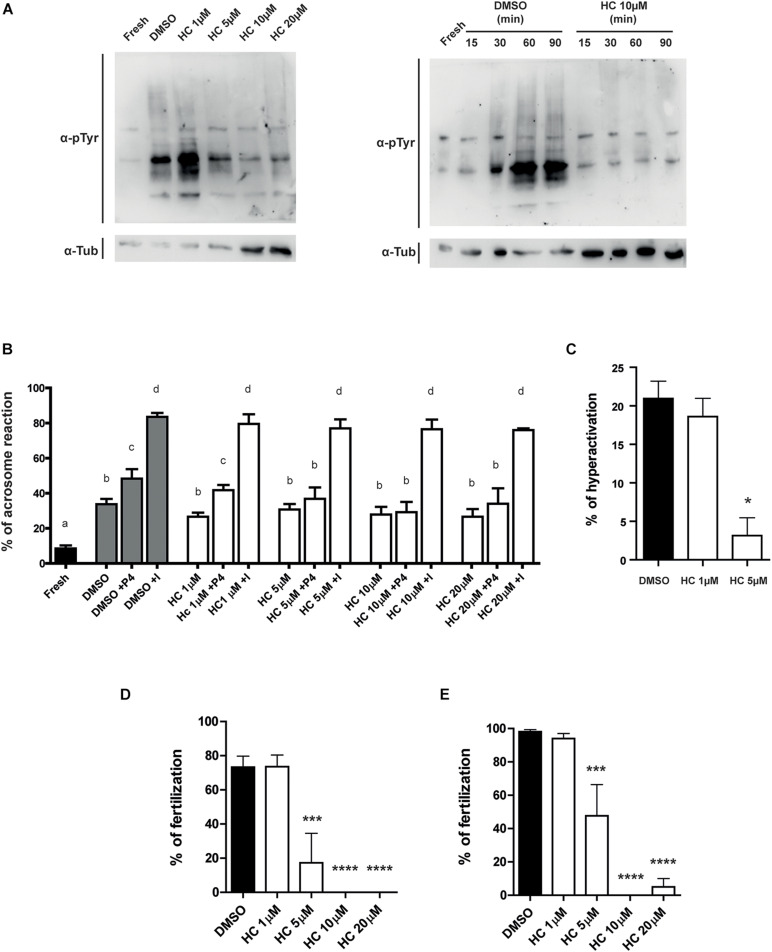
Effect of HC on capacitation-associated functional parameters and *in vitro* fertilizing ability of sperm exposed to HC during capacitation. **(A)** Protein tyrosine phosphorylation analysis for protein extracts from non-capacitated sperm (Fresh) or sperm incubated under capacitating conditions in medium containing either different concentrations of HC (1, 5, 10, and 20 μM) or DMSO (vehicle) as control (left panel). Protein tyrosine phosphorylation as a function of incubation time for non-capacitated sperm (fresh) or sperm incubated under capacitating conditions in a medium containing 10 μM HC (right panel). Samples were analyzed by Western blot using a mouse antiphosphotyrosine monoclonal antibody (α-pTyr). In all cases, a representative Western blot from at least three different experiments is shown. **(B)** Percentage of acrosome reaction determined by Coomassie Brilliant Blue staining in cauda epididymal sperm incubated under capacitating conditions for 90 min in medium containing different concentrations of HC (1, 5, 10, and 20 μM) and exposed to either progesterone (P4) or Ca^2+^ ionophore (I) during the last 15 min of incubation. Non-capacitated (fresh) sperm were used as control. Means not sharing the same letters are significantly different. **(C)** Percentage of hyperactivation evaluated by CASA for sperm incubated under capacitating conditions for 90 min in a medium containing 1 or 5 μM HC or DMSO (vehicle) as control. **(D,E)** Percentage of fertilization obtained using cauda epididymal sperm incubated for 90 min in capacitating medium containing different concentrations of HC (1, 5, 10, and 20 μM) or DMSO (control) and then coincubated with either Cumulus Oocytes Complexes (COC) for 3 h **(D)** or ZP-free oocytes for 1 h **(E)**. At the end of incubations, fertilization was evaluated. Eggs were considered fertilized when at least one decondensing sperm nucleus or two pronuclei were observed in the egg cytoplasm and the percentage of fertilized eggs was determined. Data are mean ± SEM of at least *n* = 3 independent experiments; *p < 0.05; ***p < 0.0005, and ****p < 0.0001 vs. DMSO.

Having confirmed the inhibitory effect of HC on the intracellular Ca^2+^ increase that occurs during capacitation, we then analyzed the effect of the presence of the compound during capacitation on several Ca^2+^-dependent events that occur during this process. Western blot analysis showed both that sperm exposed for 90 min to ≥ 5 μM HC failed to undergo the characteristic increase in protein tyrosine phosphorylation levels during capacitation (Figure 2A, left panel), and that this inhibitory effect was already detected at 30 min incubation in the presence of a fixed 10 μM HC concentration (minimal concentration with maximal effect) (Figure 2A, right panel). We then examined the effect of HC on the occurrence of the acrosome reaction, an event essential for fertilization. Results revealed that whereas neither the spontaneous nor the ionophore-induced acrosome reaction were affected by HC at any of the concentrations tested, exposure to the compound produced a significant decrease in the percentage of progesterone-induced acrosome reaction which was already evident at 5 μM HC (Figure 2B). Finally, based on the key role of CatSper for hyperactivation, we examined the effect of HC on the development of this vigorous sperm motility essential for egg coat penetration. Analysis of sperm motility by CASA showed that sperm incubated with 5 μM HC during capacitation produced a significant reduction in the percentage of hyperactivated cells compared with controls (Figure 2C), as well as in several parameters associated with hyperactivation (Supplementary Table 1). Higher concentrations of HC (10 or 20 μM) severely affected sperm motility preventing from reaching at least 200 motile sperm required for CASA analysis.

### Effect of HC on *in vitro* Sperm Fertilizing Ability

**FIGURE 3:**
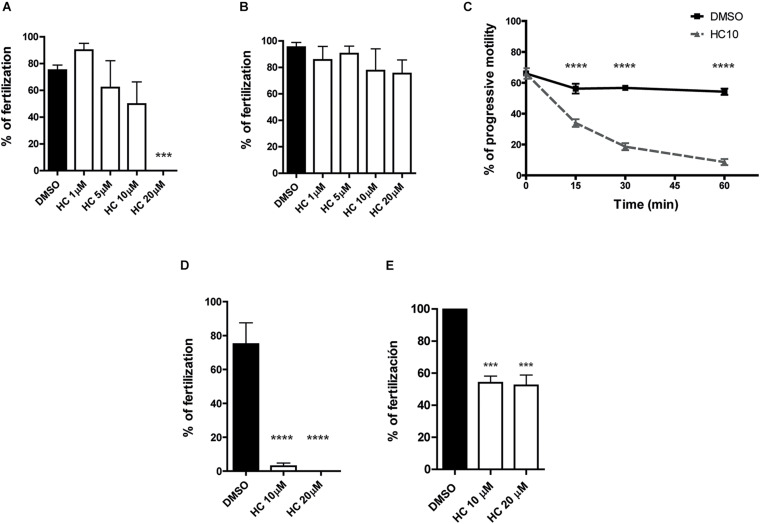
*In vitro* fertilizing ability of sperm exposed to HC after capacitation. **(A,B)** Cauda epididymal sperm incubated under capacitating conditions for 90 min in medium alone were coincubated with either COC for 3 h **(A)** or ZP-free oocytes for 1 h **(B)**, in media containing different concentrations of HC (1, 5, 10, and 20 μM) or DMSO as control. At the end of incubations, fertilization was evaluated. Eggs were considered fertilized when at least one decondensing sperm nucleus or two pronuclei were observed in the egg cytoplasm and the percentage of fertilized eggs was determined. **(C)** Percentage of sperm progressive motility as a function of coincubation time in the presence of 10 μM HC. Samples collected at different capacitation times were analyzed by light microscopy (×400). **(D,E)** Sperm incubated under capacitating conditions for 90 min in medium alone were then incubated with HC (10 and 20 μM) for 30 min prior to their coincubation with either COC for 3 h **(D)** or ZP-free oocytes for 1 h **(E)**, in media containing different concentrations of HC (10 and 20 μM) or DMSO as control. At the end of incubations, fertilization was evaluated as described above. Data are mean ± SEM of at least *n* = 3 independent experiments; ***p < 0.0005; ****p < 0.0001 vs. DMSO.

Considering the inhibitory effects of HC on sperm functional events essential for fertilization (i.e., Ca^2+^ increase, acrosome reaction, hyperactivation), we next evaluated the impact of the drug on the sperm fertilizing ability. For this purpose, sperm were exposed to different concentrations of HC during capacitation as previously described and then used for inseminating eggs either surrounded by both the cumulus oophorus and the ZP (COC) or denuded of the two coats (ZP-free eggs). Results revealed that in both cases, HC produced a significant decrease in the percentage of fertilized eggs which became evident at 5 μM HC and was severe or complete at higher concentrations (Figures 2D,E). To exclude a possible effect of the inhibitor on the eggs, ZP-free oocytes were exposed to 20 μM HC for 60 min, washed and then coincubated with capacitated sperm for 60 min. Under these conditions, no effect of HC on egg penetrability was observed (DMSO: 100% vs. HC: 100%, *n* = 3, NS). To investigate whether HC was capable of exerting its effect not just during but also after capacitation, *in vitro* fertilization assays were carried out exposing already capacitated sperm to HC just during the gamete coincubation period. Under these conditions, no significant differences were observed for either COC or ZP-free oocytes at any of the concentrations assayed except when COC were exposed to the maximum (20 μM) HC concentration (Figures 3A,B). Surprisingly, in spite of the described overall lack of effect of HC on the sperm fertilizing ability, the cells exhibited a clearly affected progressive motility at the end of gamete coincubation. Analysis of the kinetics of motility inhibition carried out by exposing capacitated sperm to a fixed concentration of HC (10 μM) revealed that motility was already significantly affected at 15 min incubation, reaching values of less than 10% at the end of incubation (Figure 3C), similar to those observed for sperm exposed to HC during capacitation (see Figure 1D). To investigate whether the lack of effect under these conditions could be due to the need of a period of exposure of sperm to the drug before they contact the eggs, capacitated sperm were preincubated with HC (10 and 20 μM) for 30 min and then coincubated with the eggs in the presence of the inhibitor as previously described. Under these conditions, a significant decrease in the percentage of fertilized eggs was observed for both COC and ZP-free oocytes (Figures 3D,E), indicating that the sperm fertilizing ability is affected by HC when either capacitating or capacitated sperm are exposed to the inhibitor before they are incubated with the eggs.

**FIGURE 4:**
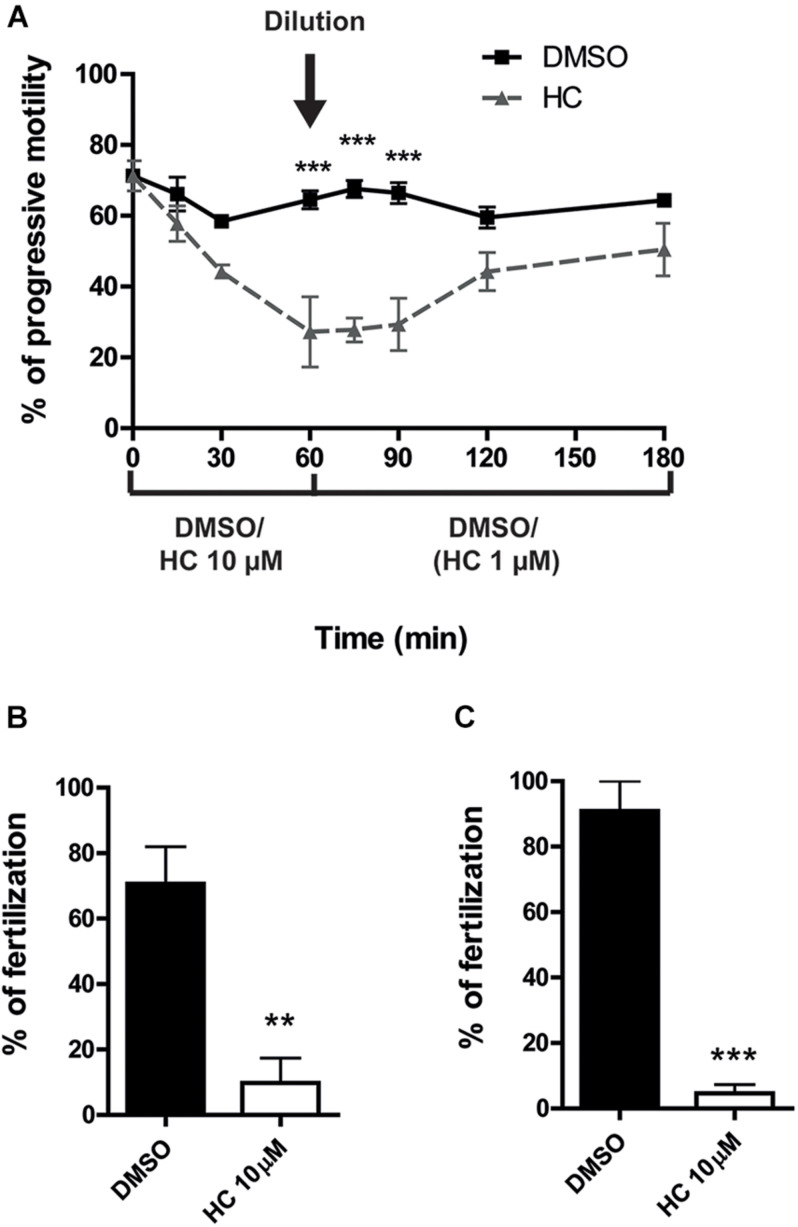
*In vitro* fertilizing ability of sperm exposed to HC before capacitation. **(A)** Percentage of progressive motility analyzed as a function of incubation time for sperm incubated for 60 min in a high, non-capacitating concentration in a medium containing 10 μM HC or DMSO as control, then diluted in medium alone to reach both a capacitation sperm concentration and a 1 μM HC concentration, and finally incubated for an additional 120 min. Samples collected at different time points were analyzed by observation under light microscopy (400x). **(B,C)** Sperm incubated under capacitating conditions (after dilution) were coincubated with COC for 3 h **(B)** or with ZP-free oocytes for 1 h **(C)**. At the end of incubations, fertilization was evaluated. Eggs were considered fertilized when at least one decondensing sperm nucleus or two pronuclei were observed in the egg cytoplasm and the percentage of fertilized eggs was determined. Data are mean ± SEM of at least *n* = 3 independent experiments; **p < 0.005; ***p < 0.0005 vs. DMSO.

The experimental approaches described above simulate the situation where a CatSper inhibitor is used for female contraception since both capacitation and gamete interaction take place in the female tract. To examine the potential use of HC for male contraception, epididymal sperm at high, non-capacitating concentrations (1–10 × 10^7^ sperm/ml), were incubated with 10 μM HC for 60 min to better emulate the exposure of sperm to the drug within the male tract. At the end of incubation, an aliquot of the suspension was diluted (1/10) in fresh medium to reach both a sperm concentration that allows capacitation (1–10 × 10^6^ sperm/ml) and a final 1 μM HC concentration known to have no effects on sperm functionality, and the cells were incubated for additional 90–120 min under these capacitating conditions. Sperm samples were then recovered at different times during the whole (180 min) incubation period to analyze their progressive motility and used for *in vitro* fertilization after 90 min capacitation. Results showed that whereas sperm progressive motility was significantly impaired during the first hour of incubation at the high, non-capacitating sperm concentration, it increased as a function of time after dilution, reaching levels not significantly different from controls 60 min later (Figure 4A). However, in spite of their motility recovery, sperm remained unable to fertilize either COC or ZP-free eggs (Figures 4B,C), revealing an irreversible effect of HC on the sperm fertilizing ability.

### Effect of HC Inhibitor on *in vivo* Sperm Fertilizing Ability

**FIGURE 5:**
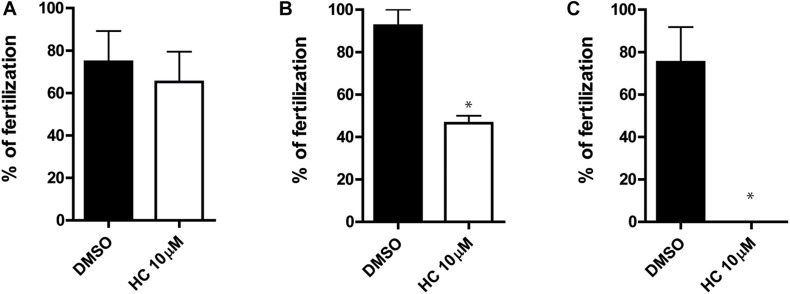
*In vivo* fertilizing ability of sperm exposed to HC before intrauterine insemination. **(A–C)** Cauda epididymal sperm in a non-capacitating concentration (1–10 × 10^7^ sperm/ml) were incubated in a medium containing 10 μM HC for 0 min **(A)**, 30 min **(B)**, and 60 min **(C)** and then inseminated in the uterus of superovulated females. After 15 h, eggs were recovered from the ampulla and the percentage of fertilized eggs was determined. Eggs were considered fertilized when at least one decondensing sperm nucleus or two pronuclei were observed in the egg cytoplasm and the percentage of fertilized eggs was determined. Data are mean ± SEM of at least *n* = 3 independent experiments; *p < 0.05 vs. DMSO.

Having confirmed that HC was capable of affecting the sperm fertilizing ability under different *in vitro* conditions, we next examined whether the compound could also inhibit the sperm ability to fertilize the eggs *in vivo.* For this purpose, caudal epididymal sperm in a high, non-capacitating concentration (10^7^–10^8^ sperm/ml) were exposed to 10 μM HC for different times (0, 30, and 60 min) as described above and then injected into one of the uterine horns of a superovulated female using a sperm suspension preincubated with DMSO in the contralateral horn as a control. After 15 h, oocytes were recovered from the ampulla and the percentages of fertilized eggs were analyzed. Results showed a significant reduction in the percentage of fertilization compared with controls for sperm preincubated with the drug for 30 min and a complete inhibition for those exposed to HC for 60 min (Figures 5A–C), confirming the ability of HC to irreversibly block fertilization under *in vivo* conditions.

## Discussion

Substantial evidence supports that sperm-specific CatSper channel is essential for male fertility (Singh and Rajender, 2015) and, thus, an attractive target for non-hormonal contraception (Ren et al., 2001; Drevet, 2018). In the present study, we show for the first time that CatSper inactivation by an efficient channel inhibitor (Carlson et al., 2009; Ernesto et al., 2015) affects both *in vitro* and *in vivo* sperm fertilizing ability, independently of the capacitation status of the cells, representing a solid proof of concept for the idea of developing a male/female contraceptive based on the pharmacological blockage of CatSper activity.

Previous results revealed that HC, identified as an effective CatSper inhibitor, significantly affects CatSper currents (Carlson et al., 2009; Ernesto et al., 2015) as well as intracellular Ca^2+^ levels in sperm perfused with the drug (Carlson et al., 2009). To examine whether HC was capable of affecting Ca^2+^ entry into capacitating cells, we first analyzed the effect of the compound on the characteristic intracellular Ca^2+^ increase that occurs during capacitation. Flow cytometry results confirmed the ability of HC to significantly affect intracellular Ca^2+^ increase in capacitating sperm without eliciting a toxic effect as judged by the normal levels of sperm viability observed throughout the incubation period. Given the inhibitory activity of HC on Ca^2+^ entry during sperm capacitation, we next investigated its effects on different capacitation-associated sperm functional events. Our observations revealed that the presence of HC during capacitation produced a significant and time-dependent decrease in sperm progressive motility previously reported to occur in KO sperm for each of the four pore-forming (CatSper 1–4) α subunits (Qi et al., 2007), supporting the effect of HC on CatSper activity as well as the involvement of CatSper not only in hyperactivation development (Carlson et al., 2003; Quill et al., 2003) but also in the progressive motility of the cells (Quill et al., 2003; Qi et al., 2007; Tamburrino et al., 2014).

Exposure of sperm to HC also produced a clear inhibition of the characteristic Ca^2+^-dependent increase in protein tyrosine phosphorylation that occurs during sperm capacitation. This result differed from the increase in protein tyrosine phosphorylation observed for CatSper KO sperm (Chung et al., 2014; Ded et al., 2020) and could be due to the reported biphasic effect of Ca^2+^ on the cAMP/tyrosine phosphorylation pathway showing that whereas the complete absence of Ca^2+^ by addition of EGTA to the media induces an increase in tyrosine phosphorylation as the one observed for CatSper KO sperm (Chung et al., 2014), the presence of small traces of the cation causes a decrease in this parameter (Navarrete et al., 2015) like that detected in the present study for HC-treated sperm.

Considering evidence indicating that Ca^2+^ influx through CatSper also initiates a tail-to-head Ca^2+^ propagation (Xia and Ren, 2009) involved in the acrosome reaction (Stival et al., 2018), we also analyzed the effect of HC on the occurrence of this exocytotic event. Of note in this regard, HC neither increases intracellular Ca^2+^ nor induces the acrosome reaction by itself as reported for several other CatSper inhibitors such as NNC-0396, mibefradil, and MDL12330A (Chávez et al., 2018). Our results indicated that although the presence of HC during capacitation did not affect the levels of either the spontaneous or ionophore-induced acrosome reaction, it significantly inhibited its induction by progesterone. Similar results were observed in mice treated with matrine, an alkaloid that also inhibits CatSper currents and produces a significant reduction in the percentages of progesterone-induced acrosome reaction (Luo et al., 2016). Whereas progesterone stimulates human but not mouse CatSper activity (Lishko et al., 2011; Strünker et al., 2011; Mannowetz et al., 2017), our observations can be explained by the reported involvement of the hormone in other cellular events (i.e., PKA activation/relocalization) that promote Ca^2+^ influx through CatSper, leading to the acrosome reaction (Stival et al., 2018).

Consistent with the essential role of Ca^2+^ and CatSper for hyperactivation development, CASA studies revealed that exposure of sperm to HC during capacitation produced a clear decrease in the percentage of hyperactivated cells which became already evident at 5 μM and which resembles the lack of hyperactivation observed in CatSper KO mice (Carlson et al., 2003; Quill et al., 2003). Taken together, our observations on the effect of HC on the different Ca^2+^ and capacitation-associated events (i.e., progressive motility, pTyr, acrosome reaction, hyperactivation) support the idea that HC produces a pharmacological phenocopy of CatSper-null sperm (Carlson et al., 2009).

In agreement with the inhibition of the different sperm functional parameters, the presence of HC during capacitation produced a severe/complete blockage of the sperm ability to fertilize COC as the one observed for CatSper mutant sperm (Ren et al., 2001; Quill et al., 2003). This was an important finding as most studies using CatSper inhibitors have not investigated their effects on the sperm fertilizing ability. Of note, these HC-treated sperm also showed clear defects to fertilize ZP-free eggs, indicating the existence of specific defects in their gamete fusion ability. This result was striking as CatSper KO sperm are able to fertilize ZP-free eggs (Ren et al., 2001; Quill et al., 2003) and could be due to the observed effect of HC on Slo3 (Navarro et al., 2007; Carlson et al., 2009), the principal sperm-specific K^+^ channel (Santi et al., 2010; Zeng et al., 2011; Brenker et al., 2014) essential for male fertility and reported to be required for ZP-free egg fertilization (Santi et al., 2010). Whereas the use of a CatSper inhibitor that does not affect Slo3 in murine sperm (i.e., RU1968) (Rennhack et al., 2018) might contribute to clarify our observations, the additional effect of HC on Slo3 might represent an advantage for contraception purposes as it could improve the blockage of the sperm fertilizing ability without increasing side effects due to Slo3 cell specificity.

Having established the effect of HC on the sperm fertilizing ability when present during capacitation, we next investigated whether the compound could exert its inhibitory effect after capacitation when present just during the gamete coincubation period. Under these conditions, HC failed to inhibit the sperm ability to fertilize either COC or ZP-free oocytes in spite of exhibiting progressive motility defects similar to those observed for sperm exposed to HC during capacitation. Whereas these results could be interpreted as indicating that HC needs to be present during capacitation in order to affect sperm functionality, exposure of already capacitated sperm to HC prior to their coincubation with the oocytes significantly inhibited the sperm ability to fertilize both COC and ZP-free oocytes, indicating that sperm function is affected by HC when either capacitating or capacitated cells are exposed to the compound before they are coincubated with the eggs. Considering that CatSper is required not only for the onset of hyperactivation but also for its maintenance (Carlson et al., 2009), it is likely that HC requires a period of time to act on sperm to either prevent the onset of hyperactivation in capacitating cells or cause the loss of hyperactivation in already capacitated cells. This might also explain why HC present just during gamete coincubation was capable of affecting COC fertilization at the highest concentration but not zona-free egg fertilization at any concentration since the longer time required for penetration of the egg coats might provide better conditions for HC to affect sperm than the rapid sperm interaction with the denuded eggs.

The results obtained using capacitating or capacitated sperm simulate the possible effect that the compound may exert if present in the female tract where both capacitation and fertilization take place. In order to explore the potential use of HC for male contraception, sperm in a high, non-capacitating concentration were exposed to HC *in vitro*, diluted to reach both a sperm concentration that allows capacitation and a drug concentration (1 μM) unable to affect the cell, and finally coincubated with the eggs in the absence of the inhibitor. Under these conditions, HC affected sperm progressive motility as observed for sperm capacitated in the presence of the inhibitor although in this case, the experimental approach revealed the reversibility of the drug effect as judged by the subsequent recovery of sperm progressive motility during the following capacitation step. Motility recovery after dilution of HC together with the normal viability of HC-treated sperm strongly supports the lack of toxic effects of the inhibitor on sperm. Moreover, as exposure of ZP-free eggs to 20 μM HC did not affect their fertilizability, it is likely that HC does not affect other cells either, a key requisite for a contraceptive approach. Interestingly, however, despite progressive motility recovery, sperm remained unable to fertilize both COC and ZP-free oocytes. Given that 1 μM HC during capacitation does not affect sperm fertilizing ability and the absence of HC during gamete coincubation, our observations indicate an effect of HC on sperm during the first step of incubation at the high, non-capacitating sperm concentration also observed when using a BSA-free incubation medium (data not shown). Whereas we cannot exclude the possibility that HC is affecting some capacitation-associated signaling pathways triggered during incubation under these conditions, our observations support the idea that HC is capable of interacting with CatSper before capacitation, leading to an irreversible blockage of the channel activity. The prevalence of sperm fertilizing defects in spite of progressive motility recovery indicates a sustained effect of HC on sperm functional events essential for fertilization (i.e., acrosome reaction, hyperactivation), consistent with the finding that HC effect on hyperactivation onset is maintained after removal of the drug (Carlson et al., 2009). Whereas the mechanisms underlying the irreversible blocking activity of HC are still unknown, it is interesting to mention recent *in silico* studies reporting that HC has a high affinity binding site inside the pore of the heterotetramer (Beltrán et al., 2020), making it possible that HC firmly binds to one or more of CatSper pore subunits. Together, our observations support that HC is capable of blocking the sperm fertilizing ability independently of the capacitation status of the cells (i.e., before, during, and after capacitation).

Finally, having observed that sperm exposed to HC were unable to fertilize the eggs under *in vitro* conditions, we evaluated whether the compound could also exert its effects upon *in vivo* fertilization. Under these conditions, we observed a significant or complete decrease in the percentage of fertilized eggs recovered from the ampulla which correlated with the decrease in sperm progressive motility observed as a function of time just before insemination. These results suggest that progressive motility defects may be responsible for fertilization impairment in the oviduct, in agreement with the need of CatSper for sperm transport within the female tract (Ho et al., 2009; Chung et al., 2014), revealing also the irreversible effect of HC on sperm function under *in vivo* conditions. To our knowledge, these findings represent the first evidence showing the irreversible inhibitory effect of a CatSper blocker on *in vivo* fertilization (Amory, 2020), a finding of particular relevance for contraception, especially considering the different molecular mechanisms underlying *in vitro* and *in vivo* fertilization (Ded et al., 2020).

**FIGURE 6:**
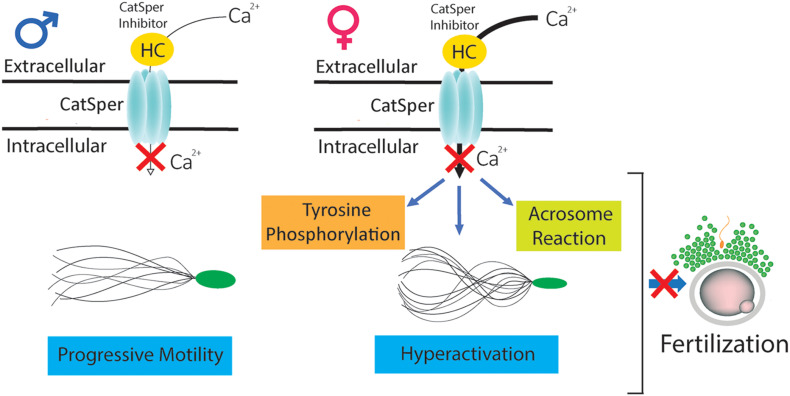
Mechanisms underlying the effects of HC on sperm fertilizing ability. CatSper inhibitor HC is capable of interacting with sperm before (in the male) as well as during or after capacitation (in the female), inhibiting calcium entry into the cells and leading to defects in several capacitation-associated functional events (i.e., tyrosine phosphorylation, acrosome reaction, hyperactivation) that finally block the sperm fertilizing ability.

In summary, our *in vitro* and *in vivo* studies indicate that HC is capable of interacting with CatSper before, during, and after capacitation, inhibiting calcium entry into the cells and leading to clear defects in several capacitation-associated functional parameters (i.e., tyrosine phosphorylation, acrosome reaction, hyperactivation) that finally block the sperm fertilizing ability (Figure 6). These results constitute a solid proof of concept for CatSper inhibition as a potential non-hormonal contraceptive approach. Moreover, the finding that HC exerts a rapid and sustained inhibitory effect upon CatSper activity independently of the capacitation status of the cells support the use of CatSper inhibitors for both male and female contraception (Lishko, 2016).

Considering the relevance of CatSper for human fertility (Brown et al., 2018) and recent findings from our group supporting the ability of HC to inhibit human CatSper activity (Brukman et al., 2019), the development of a non-hormonal contraceptive method based on CatSper inactivation represents a very attractive approach to avoid the many non-desired effects associated with the use of hormones in both women and men (i.e., hormonal imbalance, increased risk of cancer, weight gain, metabolic disturbances, etc.) or when hormones are contraindicated for health reasons. Non-hormonal contraceptives became of particular interest for men who have the condom as the only reversible contraceptive of choice and would neither require the long periods of time for reaching and for recovering from azoospermia nor the continuous need of semen analysis associated with hormonal methods.

Future research on CatSper function including *in vivo* approaches will contribute not only to meet the needs of safe, non-hormonal male/female contraceptives but also to a better understanding, diagnosis, and treatment of human infertility.

## Data Availability Statement

The raw data supporting the conclusions of this article will be made available by the authors, without undue reservation.

## Ethics Statement

The animal study was reviewed and approved by CICUAL-IBYME.

## Author Contributions

LC and GC performed most experiments with assistance from SG and VS. LC, GC, and PC designed the experiments and analyzed the results. LC, SG, and PC wrote the manuscript. All authors read, corrected, and approved the final manuscript.

## Conflict of Interest

The authors declare that the research was conducted in the absence of any commercial or financial relationships that could be construed as a potential conflict of interest.

## Supplementary Material

Supplementary Figure 1 Effect of HC on sperm progressive motility analyzed by CASA. Percentage of progressive motility evaluated by CASA for sperm incubated under capacitating conditions for 90 min in medium containing 1 or 5 μM HC or DMSO (vehicle) as control. Higher concentrations of HC (10 or 20 μM) severely affected sperm motility preventing reaching the at least 200 motile sperm required for CASA analysis. Data are mean ± SEM of at least *n* = 3 independent experiments; ^∗^*p* < 0.05 vs. DMSO.

Supplementary Table 1 Sperm motility parameters analyzed by CASA. CASA parameters analyzed for sperm incubated under capacitating conditions for 90 min in medium containing 1 or 5 μM HC or DMSO (vehicle) as control. Data are mean ± SEM of at least *n* = 3 independent experiments; ^∗^*p* < 0.05; ^∗∗^*p* < 0.005; ^****^*p* < 0.0001 vs. DMSO. *VCL*, curvilinear velocity; *VSL*, straight-line velocity; *VAP*, average path velocity; *LIN*, linearity; *STR*, straightness; *ALH*, amplitude of lateral head displacement; *BCF*, beat cross frequency.

## Abbreviations

AR: acrosome reaction
CASA: computer-assisted sperm analysis
CatSper: cation channel of sperm
COC: cumulus–oocyte complexes
HC: HC-056456
MFI: mean fluorescence intensity
P4: progesterone
PI: propidium iodide
ZP: zona pellucida.
