# Supplementary material for: Pharmacological Inactivation of CatSper Blocks Sperm Fertilizing Ability Independently of the Capacitation Status of the Cells: Implications for Non-hormonal Contraception
Source: Front Cell Dev Biol. 2021 Jul 6;9:686461. doi: 10.3389/fcell.2021.686461 (PMC8290173; doi:10.3389/fcell.2021.686461)
Supplement: Supplementary Figure 1 — Effect of HC on sperm progressive motility analyzed by CASA. Percentage of progressive motility evaluated by CASA for sperm incubated under capacitating conditions for 90 min in medium containing 1 or 5 μM HC or DMSO (vehicle) as control. Higher concentrations of HC (10 or 20 μM) severely affected sperm motility preventing reaching the at least 200 motile sperm required for CASA analysis. Data are mean ± SEM of at least n = 3 independent experiments; ∗p < 0.05 vs. DMSO. [file Data_Sheet_1.docx]

Supplementary Material

# Supplementary Figures and Tables

## Supplementary Figure


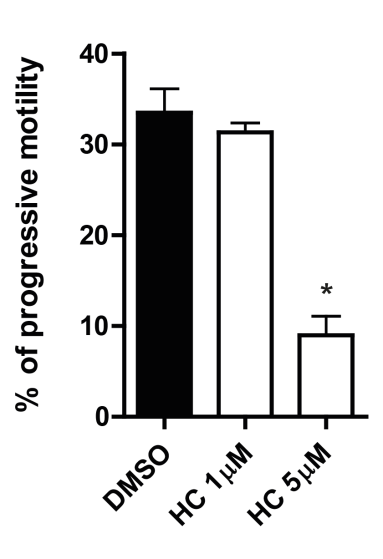


**Fig. S1. Effect of HC on sperm progressive motility analyzed by CASA.** Percentage of progressive motility evaluated by CASA for sperm incubated under capacitating conditions for 90 min in medium containing 1 or 5 μM HC or DMSO (vehicle) as control. Higher concentrations of HC (10 or 20 µM) severely affected sperm motility preventing reaching the at least 200 motile sperm required for CASA analysis. Data are mean ± SEM of at least n=3 independent experiments; **p*<0.05 vs DMSO.

## Supplementary Table

**Table S1. Sperm motility parameters analyzed by CASA.**

|  | **VCL** | **VSL** | **VAP** | **LIN** | **STR** | **ALH** | **BCF** |
| --- | --- | --- | --- | --- | --- | --- | --- |
| **Control** | 253.5±8.1 | 67.5±1.1 | 115.0±2.7 | 26.7±0.6 | 58.8±0.8 | 5.2±0.2 | 11.5±0.2 |
| **HC 1uM** | 244.6±4.2 | 63.0±1.6***** | 109.8±3.3 | 25,8±0.5 | 51.6±5.6 | 4.9±0.2 | 12.1±0.8 |
| **HC 5uM** | 169.1±35.7***** | 37.3±13.9***** | 86.6±31.3 | 21.0±3.9 | 42.9±3.5****** | 2.4±0.1******** | 9.1±1.7 |

CASA parameters analyzed for sperm incubated under capacitating conditions for 90 min in medium containing 1 or 5 μM HC or DMSO (vehicle) as control. Data are mean ± SEM of at least n=3 independent experiments; **p*<0.05, ***p*<0.005, *****p*<0.0001 vs DMSO. *VCL* (curvilinear velocity), *VSL* (straight line velocity), *VAP* (average path velocity), *LIN* (linearity), *STR* (straightness), *ALH* (amplitude of lateral head displacement), *BCF* (beat cross frequency).
